# The impact of vancomycin trough concentrations on outcomes in non-deep seated infections: a retrospective cohort study

**DOI:** 10.1186/s40360-018-0236-z

**Published:** 2018-07-31

**Authors:** Michael Wan, Sandra A. N. Walker, Elaine Martin, Marion Elligsen, Lesley Palmay, Jerome A. Leis

**Affiliations:** 1 Department of Pharmacy, Sunnybrook Health Sciences Centre, 2075 Bayview Avenue, Toronto, ON M4N 3M5 Canada; 2 Leslie Dan Faculty of Pharmacy, University of Toronto, 144 College Street, Toronto, ON M5S 3M2 Canada; 3 Division of Infectious Diseases, Sunnybrook Health Sciences Centre, 2075 Bayview Avenue, Toronto, ON M4N 3M5 Canada; 4 Sunnybrook Research Institute, Sunnybrook Health Sciences Centre, 2075 Bayview Avenue, Toronto, ON M4N 3M5 Canada; 5 Department of Medicine, Sunnybrook Health Sciences Centre, 2075 Bayview Avenue, Toronto, ON M4N 3M5 Canada; 6 Faculty of Medicine, University of Toronto, 1 King’s College Circle, Toronto, ON M5S 1A8 Canada; 7 Present address: Elaine Martin, Trillium Health Partners, 100 Queensway W, Mississauga, ON L5B 1B8 Canada

**Keywords:** Vancomycin, Non-deep seated infections, Trough concentrations, Levels, Therapeutic drug monitoring, Outcomes

## Abstract

**Background:**

Guidelines recommending vancomycin trough concentrations > 10 mg/L in non-deep seated infections are based on expert opinion. The objective of this study was to evaluate patients with non-deep seated infections treated with short-course vancomycin to determine whether there were differences in outcomes with trough concentrations of ≤10 mg/L (low) versus > 10 mg/L (high).

**Methods:**

A retrospective cohort study of patients hospitalized between March 10, 2010 and December 31, 2015 who received ≤14 days of vancomycin to treat a non-deep seated infection and had at least one steady state trough concentration was completed. Patient data for the low versus high trough cohorts were compared using appropriate statistical tests and binary logistic regression was used to identify factors associated with clinical outcome.

**Results:**

Of 2098 patients screened, 103 (5%) met inclusion criteria. Baseline characteristics between cohorts were not different. Clinical cure was not different between the low (42/48 [88%]) and high trough (48/55 [87%]) cohorts (*p* > 0.99) and vancomycin trough concentration was not associated with clinical outcome (*p* = 0.973). More patients in the high trough group had dosing changes (7/48 [15%] vs. 22/55 [40%], *p* = 0.0046), with approximately three times more dose adjustments per patient (0.17 vs. 0.55, *p* = 0.0193). No signal for increased vancomycin resistance associated with vancomycin troughs was identified.

**Conclusions:**

No difference in clinical or microbiological outcomes based on vancomycin trough concentrations were observed in patients with non-deep seated infections treated with vancomycin for ≤14 days. Targeting higher vancomycin trough concentrations of > 10 mg/L may be associated with increased workload with no corresponding benefit in clinical or microbiological outcomes in these patients.

## Background

Vancomycin was discovered over 60 years ago; however, it was not until the early 1980s that its clinical use sharply increased in response to a rise in the worldwide prevalence of methicillin-resistant *Staphylococcus aureus* (MRSA) [1]. Unfortunately, controversy regarding the optimal dosing and pharmacokinetic/pharmacodynamic targets continues to plague the use of vancomycin [2]. Current guidelines recommend targeting vancomycin serum trough concentrations of 15–20 mg/L for complicated infections (e.g. endocarditis, osteomyelitis, pneumonia, meningitis, etc.), and suggest maintaining a trough of > 10 mg/L for all patients (including those with non-deep seated infections with a planned duration of ≤14 days) to avoid development of resistance to vancomycin (Quality and Grade of Evidence for each recommendation in the 2009 IDSA guidelines was IIIB) [3–5].

The emergence of vancomycin resistance has not been observed in patients treated with short term vancomycin (≤14 days) for the management of non-deep seated infections (e.g. bacteremia when endocarditis or other non-cardiac seeding [e.g. bone or brain] has been ruled out; uncomplicated skin and soft tissue infections; and urinary tract infections in the absence of anatomic abnormalities, renal stones, or renal abscess).

Clinical evidence to support the need to maintain vancomycin serum trough concentrations above 10 mg/L for management of non-deep seated infections is lacking and the practice of targeting higher trough concentrations has obvious downsides. This practice increases the complexity of the dosing and monitoring of vancomycin, results in additional blood draws from the patient, increases the workload for the clinical team, and may therefore, increase the risk of medical error and harm to the patient. We hypothesize that there is no difference in clinical or microbiological outcomes associated with vancomycin trough concentrations of ≤10 mg/L versus > 10 mg/L when vancomycin is used in the setting of non-deep seated infections for a period of ≤14 days.

The primary objective of this study was to evaluate patients with non-deep seated infections who were treated with vancomycin for a period of ≤14 days to determine whether there were differences in clinical outcome with serum trough concentrations of vancomycin of ≤10 mg/L versus > 10 mg/L. The secondary objectives of this study were to identify factors which may affect clinical outcome, evaluate safety outcomes (kidney injury), assess workload based on dose adjustment(s), and identify changes in microbial resistance during the vancomycin treatment course.

## Methods

### Study design

A retrospective chart review of eligible patients was conducted. Patients were identified for eligibility using the Stewardship Program Integrating Resource Information Technology (SPIRIT) database of our Antimicrobial Stewardship Program [6]. A SPIRIT query generated a list of inpatients at SHSC between March 12th, 2010 and December 31st, 2015, inclusive, with at least one steady state vancomycin trough concentration. Hospital charts for the patients identified by this query were retrieved from Health Data Resources (HDR) and reviewed to confirm patient eligibility for this study.

### Patient eligibility

Adult inpatients (aged ≥18 years) were eligible for study inclusion if they were admitted to SHSC between March 12th, 2010 and December 31st, 2015, received a minimum of 48 h and a maximum of 14 days of vancomycin for a presumed or confirmed non-deep seated infection (defined below) associated with any bacteria for which vancomycin may be indicated, and for whom at least one steady state vancomycin trough concentration was available. A steady state vancomycin trough concentration was defined as a level obtained at the earliest prior to the 3rd dose for ≥ every 12 h dosing, and prior to the 4th dose for ≤ every 8 h dosing; or a random vancomycin concentration obtained at least 24 h after vancomycin initiation in patients receiving continuous infusion. An exception was made for inclusion of patients receiving a maximum of 15 days of vancomycin, if the treating physician’s intent was to treat for 14 days. Patients were eligible for inclusion if their infection-related diagnosis was:

- Uncomplicated skin and soft tissue infections (SSTI): folliculitis, carbuncle(s), surgical site infections, wound infections, non-suppurative cellulitis or erysipelas;
- Urinary tract infections (UTI) in the absence of anatomic abnormalities, renal abscess, or renal stones;
- Bacteremia without any evidence of seeding to heart, bone/joint, brain, or lungs; or
- Coagulase negative *Staphylococci* (CNST) line-related infections (excluding tunnel infection or vascular graft infections).

If patients received more than one course of vancomycin during their hospital stay, only data from their first vancomycin course was included, with documentation of any subsequent courses of vancomycin within 14 days of vancomycin discontinuation or patient readmission within 30 days of completion of their initial vancomycin treatment course. Subsequent vancomycin treatment courses and patient readmission were considered markers of potential relapsing infection (clinical failure) with a risk of development of a vancomycin resistant bacterial isolate. Patients were excluded if there was a presumed or documented diagnosis of abscess at any site, endocarditis, meningitis, osteomyelitis, joint infection, febrile neutropenia, pneumonia, or sinusitis; were switched to an alternate antibiotic on day 2 or 3 of vancomycin due to culture and sensitivity results; received antibiotics for no documented infection (e.g. prophylactic antibiotics); received renal replacement therapy; or if vancomycin was discontinued as a result of a patient care plan that was changed to palliative.

### Data collection and definitions

Required patient clinical and laboratory data were extracted from the hospital’s electronic databases (SPIRIT, electronic patient records [EPR/Sunnycare]) and patient medical records (HDR/Sovera) and entered into a Microsoft Excel workbook. Patients were stratified to vancomycin low or high trough cohorts based on their final documented vancomycin steady-state trough concentration (≤10 mg/L or > 10 mg/L, respectively). The primary outcome of this study was clinical cure, defined as all of the following:

- Resolution of all presenting signs and symptoms of infection within ≤14 days therapy with vancomycin; whereby patients could be discharged home on a short course of vancomycin (total duration ≤14 days) with documentation of improvement during hospital stay;
- Maintained resolution of all presenting signs and symptoms of infection for 14 days following vancomycin discontinuation;
- No additional course of antibiotics within 14 days with the same indication as initial vancomycin treatment course;
- No documentation of hospital readmission requiring antibiotic therapy within 30 days of completion of their initial vancomycin treatment course.

Secondary outcomes:

- Identification of clinical, demographic or microbiological factors associated with clinical cure;
- Kidney injury outcomes as per RIFLE (Risk, Injury, Failure, Loss of kidney function, and End-stage kidney disease) criteria [7];
- Workload based on number of dose adjustment(s) during the vancomycin course; and
- Development of microbiological resistance during the vancomycin treatment course or within 30 days of completion of the initial vancomycin treatment course.

Severity of illness was measured with the Acute Physiology and Chronic Health Evaluation (APACHE) II score for intensive care unit (ICU) patients and Pitt Bacteremia score for ward patients [8, 9]. As there are no other validated measures of severity of illness in hospital ward patients, the Pitt Bacteremia score was used in these patients, recognizing that the Pitt Bacteremia score has only been validated in patients with bacteremia. Hospital location (ICU versus ward) at time of vancomycin initiation was identified and ICU patients included those with admission or transfer to a critical care bed within 48 h of vancomycin initiation. Concomitant antibiotics were defined as any antibiotic whose administration overlapped that of vancomycin by at least 48 h and was administered for at least 48 h. The time required for each vancomycin dose adjustment was estimated from typical nursing, physician, pharmacist and pharmacy technician practices at our institution (Appendix).

### Sample size calculation

The literature was reviewed to provide an estimate of the vancomycin treatment failure rate in non-deep seated infections. Although three studies provided a treatment failure rate in SSTIs (ranging from 12 to 38% for all organisms; and 17–35% for MRSA only), the SSTIs included in these studies were complicated skin and soft tissue infections (cSSTIs) that involved deep soft tissue or required significant surgical intervention (infected ulcers, burns, and major abscesses) [10–12]. No reliable treatment failure estimates were available for UTIs or isolated uncomplicated bacteremia associated with bacteria for which vancomycin may be used. Since only patients with non-deep seated infection(s) were eligible for this study, the treatment failure rates were predicted to be lower than those reported in the literature for cSSTIs.

Based on the only available published literature, we estimated our rate of treatment failure to be between 10 and 25%; 10% being a hypothesized estimate, and 25% being the midpoint of treatment failure rates observed in the literature for cSSTIs. Thus, using a standard sample size equation for dichotomous data, 93 to 348 patients per group (based upon treatment failure rates of 10 to 25% in the high trough cohort) would be required to detect a minimal difference in failure (∆) of 10 percentage points between patients with a trough of ≤10 mg/L versus > 10 mg/L (2-tailed, *p* = 0.05, power = 0.80).

### Statistics & data analysis

Comparison of the study cohorts for interval, nominal and ordinal data were analyzed using GraphPad InStat (version 3.05, 32-bit for Win95/NT; GraphPad Software Inc., La Jolla, California). Nominal data were expressed as total number (proportion) of patients; and low trough and high trough groups were compared using the Fisher Exact test (odds ratio, 95% confidence interval and *p*-value). Interval data were expressed as mean ± standard deviation (and range) and the Kolmogorov Smirov test was used to evaluate the data for normality. If interval data passed the test for normality and standard deviations were not significantly different then a two-sided unpaired t-test was used to compare the study cohorts. If interval data were normally distributed, but standard deviations between groups were significantly different then the cohorts were compared using the two-sided unpaired t-test with Welch correction. If interval data did not pass the test for normality then a two tailed Mann-Whitney test was used. A *p*-value of < 0.05 was considered as statistically significant.

A Pearson’s Correlation matrix (univariable analysis) (SPSS version 13.0 for Windows, created September 1, 2004) was completed to identify patient clinical, microbiological, laboratory and vancomycin dosing related parameters (independent variables) associated with clinical cure (dependent variable). Patient related parameters that were evaluated by univariable analysis as independent variables were: sex, age, hospital location, length of stay at vancomycin initiation, comorbidities, immunosuppressive medications, nephrotoxic medications, severity of illness, baseline serum creatinine, final serum creatinine, serum creatinine change, risk of renal injury, kidney injury, Gram positive bacterial species identified in culture, and infectious diagnosis for which vancomycin was prescribed. Vancomycin dosing related factors that were evaluated by univariable analysis were initial dose, final dose, initial steady state vancomycin trough, final steady state vancomycin trough, and high versus low trough cohort. Any independent variables that were available for > 20% of patients and had a *p* value < 0.05 with Pearson’s Correlation were maintained in the multivariable analysis of binary logistic regression (SPSS version 13.0 for Windows, created September 1, 2004) to identify the existence of a statistically significant model (*p* < 0.05) in which all independent variables remaining in the model had an odds ratio of > 1.

## Results

**Fig. 1:**
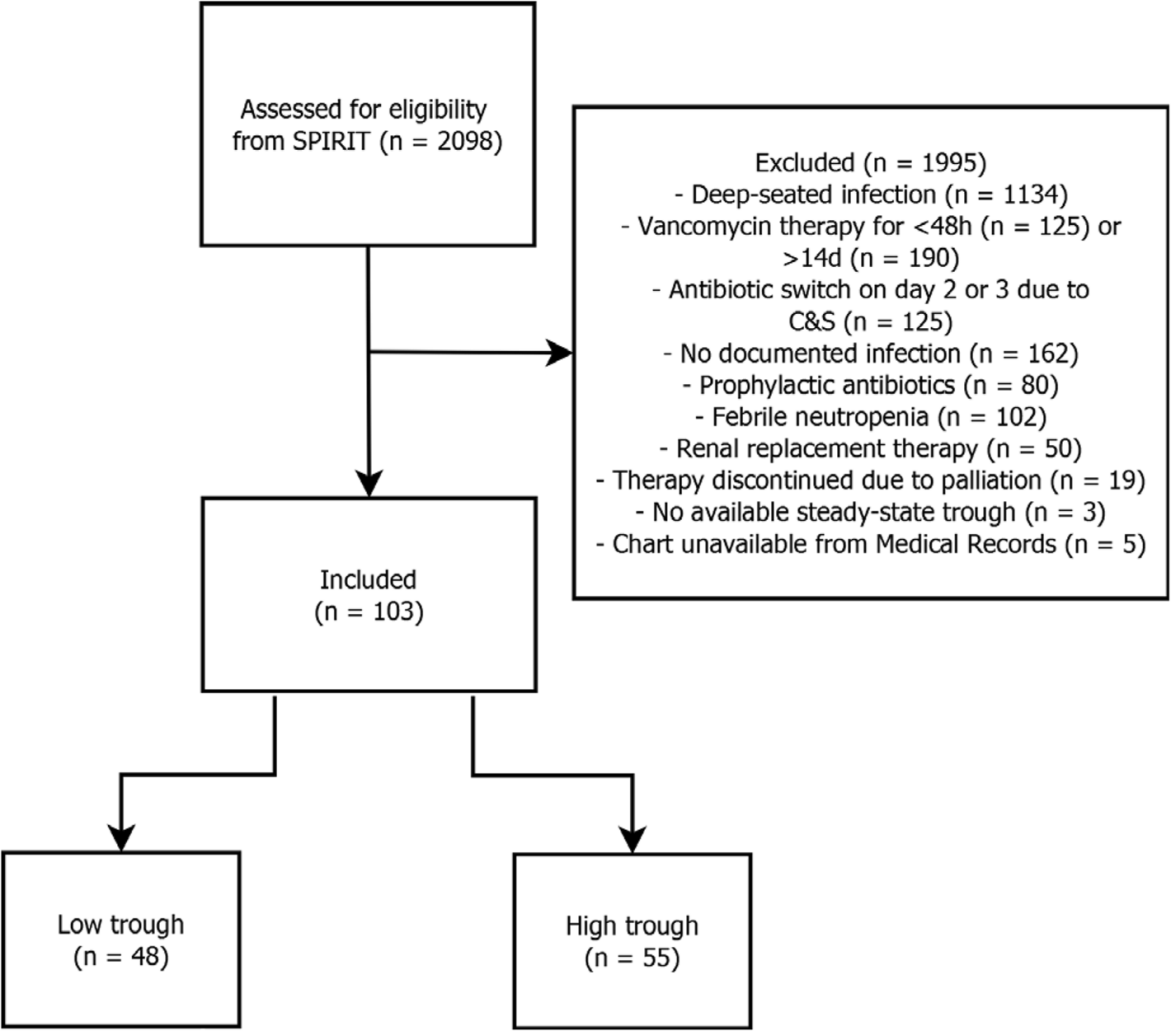
Inclusion and exclusion of flow diagram

A total of 2098 patients on vancomycin during the study period were identified from the SPIRIT database query for screening, and 103 (5%) met inclusion criteria for the study (Low trough cohort: *n* = 48, High trough cohort: *n* = 55) (Fig. 1). Of the 1995 excluded patients, the most common reason for exclusion was the presence of a deep-seated infection (1134 patients). The two groups were well-balanced in terms of baseline demographic, clinical and microbiological factors (Table 1).

**Table 1:** Patient Characteristics (*N* = 103)

	Low Trough (*N* = 48)	High Trough (*N* = 55)	Odds Ratio	95% Confidence Interval	*p*-value
Gender (Male)	27 (56%)	28 (51%)	1.24	0.57–2.70	0.69
Age on Admission (Years), mean ± SD (Range)	59 ± 19 (21–91)	67 ± 21 (19–96)			0.06
Hospital Location at Vancomycin Initiation (Ward)	38 (79%)	44 (80%)	0.95	0.36–2.48	> 0.99
Length of Stay at the time of Vancomycin Initiation (Days),median^a^(Range)	2(0–70)	2(0–187)			0.89
Any Comorbidity^b^	19 (40%)	25 (45%)	0.79	0.36–1.72	0.56
• Congestive Heart Failure	3 (6%)	8 (15%)	0.39	0.098–1.57	0.21
• Chronic Obstructive Pulmonary Disease	2 (4%)	7 (13%)	0.30	0.06–1.51	0.17
• Diabetes Mellitus	7 (15%)	10 (18%)	0.77	0.27–2.21	0.79
• Immunosuppression due to disease or drug^c^	8 (17%)	6 (11%)	1.63	0.52–5.10	0.57
APACHE II^d^, mean ± SD (ICU patients)	17 ± 6 (11–25)	23 ± 11 (6–41)			0.32
Critically Ill Ward Patients (Pitt Bacteremia Score ≥ 4)	1 (2%)	4 (7%)	0.27	0.03–2.52	0.37
Baseline Creatinine (mmol/L)	77 ± 32 (23–185)	83 ± 45 (18–254)			0.42
Use of Concomitant Nephrotoxins^e^	35 (73%)	36 (65%)	1.42	0.61–3.31	0.52
• # of Concomitant Nephrotoxins^a^ (median)	1 (0–3)	1 (0–4)			0.49
Use of Concomitant Antibiotics^f^	21 (44%)	17 (31%)	1.74	0.78–3.90	0.22
• Same Indication as Vancomycin^g^	18 (86%)	14 (82%)	1.29	0.22–7.37	> 0.99
Included Infections					
• All SSTIs	31 (65%)	32 (58%)	1.31	0.59–2.91	0.55
o Cellulitis	25 (52%)	22 (40%)	1.63	0.75–3.57	0.24
o Wound/Surgical Site Infection	6 (12%)	10 (18%)	0.64	0.21–1.92	0.59
• All UTIs	11 (23%)	7 (13%)	2.04	0.72–5.77	0.20
o MRSA UTI	2 (4%)	1 (2%)	2.35	0.21–26.75	0.60
o Enteroccocal UTI	9 (19%)	6 (11%)	1.88	0.62–5.75	0.28
• All Bacteremias	4 (8%)	10 (18%)	0.41	0.12–1.40	0.16
o CNST Bacteremia^h^	3 (6%)	8 (15%)	0.39	0.10–1.57	0.21
• Any Positive CNST in Blood	5 (10%)	14 (25%)	0.34	0.11–1.03	0.07
Microbiology					
• Patients With Positive Non-Screening Cultures for Resistant Gram positive isolates^i^	10 (21%)	22 (40%)	0.39	0.16–0.95	0.054
o Patients with MRSA Clinical Culture	2 (4%)	5 (9%)	0.43	0.08–2.35	0.44
o Patients with CNST Clinical Culture	8 (17%)	17 (31%)	0.45	0.17–1.16	0.11
• MRSA-colonized Patients	2 (4%)	8 (15%)	0.26	0.05–1.27	0.10
• VRE-colonized Patients	1 (2%)	0	3.50	0.14–88.15	0.47

*ACEI* Angiotensin converting enzyme inhibitor, AIDS Acquired immune deficiency syndrome, *APACHE* Acute Physiology and Chronic Health Evaluation, *ARB* Angiotensin II receptor blocker, *ASA* Acetylsalicylic acid, *HCTZ* Hydrochlorothiazide, *CNST* Coagulase-negative *Staphylococci*, *HIV* Human immunodeficiency virus, *ICU* Intensive Care Unit, *NSAID* Nonsteroidal anti-inflammatory drug, *PPI* Proton pump inhibitor, *SD* Standard deviation, *SSTI* Skin and soft tissue infection, *TNF-α* Tumor necrosis factor alpha, *UTI* Urinary tract infection, *VRE* Vancomycin-resistant *Enterococci*

^a^For non-normally distributed data, the median with range was reported

^b^Patients may have had more than 1 comorbidity, thus totals for specific comorbidities sum to a value greater than the number of patients with any comorbidity

^c^Disease: HIV/AIDS, asplenia, hematological malignancies, transplantation. Drug: Corticosteroids (prednisone > 5 mg/day, chemotherapy, TNF-α inhibitors, transplant medications)

^d^Arterial blood gases were not available for all ICU patients and APACHE II scoring could not be completed for these patients; reported values are based on 6 patients in low trough cohort and 7 patients in high trough cohort

^e^NSAIDs, ACEIs, ARBs, cyclosporine, tacrolimus, acyclovir, aminoglycosides, amphotericin, colistin, indinavir, adefovir, cidofovir, tenofovir, chemotherapy (e.g. carmustine, semustine, cisplatin, methotrexate, mitomycin), foscarnet, contrast dye, zoledronate, loop diuretics, HCTZ, triamterene, hydralazine, interferons, PPIs, sulfonamides, lithium, aristocholic acid, acetaminophen at > 1 g/day for > 2 years, ASA at > 1 g/day for > 2 years)

^f^Concomitant antibiotic defined as: ≥48 h overlap with vancomycin and administered for ≥48 h

^g^Denominator for percentage calculations is the number of patients (n) on concomitant antibiotics (i.e. low trough *n* = 18; high trough *n* = 14)

^h^Defined as ≥2 positive blood cultures on the same day

^i^Methicillin Resistant *Staphylococcus aureus*, Coagulase-negative S*taphylococci*, or Vancomycin Resistant E*nterococci* cultured from 3 days prior to initiation or during vancomycin course of therapy and includes a single positive culture for CNST

There was no significant difference in clinical cure (88% vs. 87%, OR 1.02, 95% CI 0.32–3.28, *p* > 0.99) or survival (100% vs. 98%, OR 2.67, 95% CI 0.11–67.13, *p* > 0.99) between the low trough and high trough groups (Table 2). One patient passed away in the high trough group for a reason unrelated to infection. Two patients in the high trough group (4%) and no patients in the low trough group (0%) were re-admitted within 30 days for the same indication as the original course of vancomycin. The 95% confidence intervals around clinical cure in the low trough versus high trough cohorts were 78–97% versus 78–96%, which completely overlap to support the finding of no significant difference in clinical cure. To detect a minimum difference in treatment failure of 1%, a sample size of 17,370 patients per group would be required. To detect a minimum difference in treatment failure of 0.5%, a sample size of 68,850 patients per group would be required. A Monte Carlo Simulation (Oracle Crystal Ball, build 11.1.4100 on 12/23/2014, 32-bit) (MCS) of 1 million iterations, using a binomial distribution, with the study observed probability of cure (Low trough = 0.88, High trough = 0.87), was conducted to determine the probability that clinical cure could theoretically be ≥10% better in the high trough cohort compared to the low trough cohort. This probability was determined to be 3%.

**Table 2:** Results (*N* = 103)

	Low Trough (*N* = 48)	High Trough (*N* = 55)	Odds Ratio	95% Confidence Interval	p-value
Clinical Outcomes					
• Clinical Cure (Primary Outcome)	42 (88%)	48 (87%)	1.02	0.32–3.28	> 0.99
• Survival	48 (100%)	54 (98%)	2.67	0.11–67.13	> 0.99
• Readmission within 30 days for same indication as original course of vancomycin	0 (0%)	2 (4%)	0.22	0.01–4.71	0.50
Microbiological Outcome					
• Patients With Positive Non-Screening Cultures for Resistant Gram positive Isolates^a^ identified up to 2 weeks after discontinuation of vancomycin	0	0	–	–	–
Vancomycin Use and Dosing					
• Final Total Daily Dose, median^b^ (mg) (Range)	2000 (500–3000)	2000 (666–4500)			0.46
• Number of Patients With Daily Dose ≥3 g/day^c^	4 (8%)	14 (25%)	0.27	0.08–0.88	0.04
• Initial Steady State Trough (mg/L)^d^	7.13 ± 2.24	12.20 ± 5.30			<0.0001^e^
• Final Steady State Trough (mg/L)^d^	7.38 ± 1.95	16.56 ± 6.56^f^			< 0.0001^f^
• Duration of Vancomycin Therapy (days)^g^	7 ± 3	7 ± 3			0.73
• # Patients Requiring Dose Adjustment	7 (15%)	22 (40%)	0.26	0.10–0.67	0.005
• # of Dose Adjustments Per Patient^b^	0.17 ± 0.43	0.55 ± 0.79			0.02^f^
• Time Estimate for Dose Adjustments Per Patient (minutes)^b^	9 ± 23	29 ± 42			0.02^f^
Renal Function Outcomes					
• Final Serum Creatinine (mmol/L)	71 ± 28	83 ± 46			0.11^e^
• % Change in Serum Creatinine	−5 ± 18	3 ± 30			0.13^e^
• At Risk for Kidney Injury^h^	0 (0%)	4 (7%)	0.12	0.01–2.25	0.12

^a^Methicillin Resistant *Staphylococcus aureus*, Coagulase-negative S*taphylococci*, or Vancomycin Resistant E*nterococci*

^b^For non-normally distributed data, the median was reported rather than the mean ± standard deviation, with the exception of: # of Dose Adjustments Per Patient, and Time Estimate for Dose Adjustment Per Patient

^c^No patients in the low trough group and 2 patients in the high trough group received ≥4 g vancomycin per day

^d^Initial vs. Final steady state trough concentration comparisons within groups: Low trough *p*-value = 0.5607 (equal SD, unpaired t-test); High trough *p*-value = 0.0009 (unequal SD, unpaired t-test Welch Corrected)

^e^Unequal SD, unpaired t-test Welch Corrected

^f^Did not pass test for normality, two tailed Mann-Whitney U test used

^g^Patients were included if the intent was to treat for ≤14 days (2 patients in the high trough group were treated for 15 days)

^h^≥50% increase from baseline serum creatinine as per RIFLE Criteria [5]

The average final steady state vancomycin troughs were 7.38 ± 1.95 mg/L and 16.56 ± 6.56 mg/L in the low and high trough groups, respectively, and the median duration of therapy in both groups was 7 ± 3 days. Although the median total daily dose between groups was similar (2 g per day in both groups), patients in the high trough group were more likely to receive 3 or more grams of vancomycin per day (*p* = 0.04) and had a significant increase from initial to final vancomycin troughs (*p* = 0.0009). Patients in the high trough group had more vancomycin dosing changes (*p* = 0.02), which in turn translated to a three-fold higher investment of health care worker time related to vancomycin therapy per patient in the high trough group (p = 0.02) (Table 2). Renal function outcomes were similar between the two groups, with no significant difference in change in serum creatinine (*p* = 0.13) and number of patients at risk for kidney injury (*p* = 0.12). However, it is noteworthy that while no patient in the low trough group was at risk for kidney injury, 4 patients in the high trough group were at risk for kidney injury (Table 2).

Univariable analysis identified five independent variables that were significantly associated with clinical outcome; however, none of these variables remained significant with multivariable analysis (Table 3). Notably, low/high trough categorization was not significantly associated with clinical outcome in patients with a non-deep seated infection with univariable analysis. The patient to variable ratio for the multivariable analysis was 21:1.

**Table 3:** Univariable and Multivariable Analyses to Identify Factors Associated with Clinical Outcome

Independent Variables	Univariable (Pearson’s Correlation)		Multivariable (Binary Logistic Regression)	
	Correlation	*P*-value	Odds Ratio	*P*-value
Low/High Trough Categorization	−0.003	0.973	–	–
Length of Stay at Vancomycin Initiation	−0.259	0.008	0.988	0.554
Heart Failure	−0.247	0.012	2.513	0.319
Pitt Bacteremia Score ≥ 4	−0.322	0.001	4.22	0.256
% Change in Serum Creatinine	−0.201	0.042	3.139	0.538
At Risk for Kidney Injury	−0.378	< 0.0001	23.606	0.163

## Discussion

This retrospective study evaluated whether differences in clinical cure exist when targeting trough concentrations ≤10 mg/L versus > 10 mg/L in patients with non-deep seated infections treated with vancomycin for ≤14 days; and, to the best of our knowledge, is the first study to do so. Although limited by a small sample size, the study enrollment reflects the entire population of patients with non-deep seated infections treated with vancomycin at our hospital between March 12th, 2010 and December 31st, 2015, inclusive. Strict eligibility criteria for study entry were used and the cohorts were well balanced for all baseline characteristics. We did not measure any difference in clinical cure with higher vancomycin trough concentrations and there was no signal for selection of resistant Gram positive bacteria in patients with a non-deep seated infection receiving ≤14 days of vancomycin. However, there was a three-fold increase in healthcare personnel workload, which introduces unnecessary complexity (increased pharmacist, pharmacy technician, nursing and physician time involvement, additional blood work, and risk of medication error with more frequent dose adjustments).

In current infectious diseases guidelines, the rationale for maintaining trough concentrations above 10 mg/L is to prevent the development of resistance; emergence of vancomycin resistance in patients treated with ≤14 days of vancomycin has never been reported in the literature [3–5]. We did not observe the emergence of any resistance to vancomycin in our study, and no patients with low vancomycin levels (< 10 mg/L) required re-admission for recurrent infection; this is a reassuring finding, since one reason for recurrent infection may be the emergence of vancomycin resistance. Vancomycin resistance in *Staphylococcus aureus* with low vancomycin levels for ≤14 days has only been observed in vitro with purposeful selection; and clinically in patients who received prolonged vancomycin exposure (6–18 weeks) [2, 13–23]. No association between vancomycin trough concentrations and emergence of vancomycin resistance has been observed for any other bacteria (e.g. CNST, *Enterococci*, other Gram positive organisms) for which vancomycin may be indicated.

As a single-center, retrospective analysis, our study has several limitations. To maximize our sample size, we screened all potential patients since the inception of our Antimicrobial Stewardship database until study closing. Despite our efforts, our final sample size was only sufficient to detect a difference of 25% points between the two groups for our primary outcome; we set a difference of 10% between the two groups as being clinically important to identify in our sample size calculation, and observed a difference of less than 1% (non-rounded clinical cure rates in low versus high trough cohorts 87.5% vs. 87.3%) between the cohorts. To be adequately powered for a difference in treatment failure of 1% or 0.5%, a sample size of 17,370 or 68,850 patients per cohort, respectively, would be necessary. Since a difference of 1% in treatment failure would not be considered clinically important in non-deep seated bacterial infections, this point illustrates the futility for the need to increase the sample size of our study and minimizes any argument that insufficient sample size may have biased against seeing a difference in clinical outcomes between the low and high trough cohorts. To further address the risk of a type II error, we conducted a MCS of 1 million iterations, using a binomial distribution, with the study observed probability of cure (Low trough = 0.88, High trough = 0.87) and found only a 3% probability that clinical cure in the high trough cohort could theoretically be ≥10% better than the low trough cohort. This result further supports the validity of the study findings that clinical cure was not significantly different between cohorts. Patients that were treated with vancomycin for less than 48 h were excluded to minimize the risk of including patients in whom infection was not considered by the physician. Although this exclusion may introduce selection bias due to early mortality, early death attributable to infection would not be anticipated with non-deep seated infections.

Due to the retrospective nature of this study, unidentified confounding factors may exist, and we made assumptions in the definition of steady state levels, as well as the definition of clinical cure and failure. We were unable to calculate the APACHE II score for all ICU patients, as not all patients had arterial blood gases available; we also extrapolated the use of the Pitt Bacteremia score to all hospital ward patients, as there are no other validated measures of severity of illness in this patient population. However, it is reassuring that both groups were well-balanced for all identified demographic, clinical and microbiological factors, including the number of patients with concomitant antibiotics, as well as the number of patients with concomitant antibiotics for the same indication as vancomycin. Only 5% of patients on vancomycin were eligible for study inclusion, primarily because most patients had deep-seated infections. Therefore, the non-deep seated infection patient population may constitute a small portion of those patients placed on vancomycin, but nevertheless, avoidance of unnecessary increases in vancomycin dosing to provide higher trough concentrations in these patients is an important quality improvement initiative to potentially reduce medication errors associated with frequent dose changes.

## Conclusions

To our knowledge, this is the first study to evaluate clinical and microbiological outcomes associated with trough concentrations in patients with non-deep seated infections treated with short course vancomycin. Our results show an increase in workload with no corresponding clinical benefit, and no signal for increased risk of resistance with vancomycin trough concentrations ≤10 mg/L for short term therapy (≤ 14 days) in non-deep seated infections.

## Appendix

### Time Estimate for Vancomycin Dose Adjustment

- Ordering vancomycin levels: 5 min
- Checking vancomycin level in Electronic Medical Record: 1 min
- Verify correct timing of dosing and levels in Medication Administration Record: 10 min
- Pharmacokinetic calculations: 10 min
- Contact physician for recommendation, obtain telephone order, write telephone order and inform nurse of new medication order: 10 min
- Pharmacist order entry: 2 min
- Preparation of new vancomycin minibag by pharmacy technician: 10 min
- Delivery of new vancomycin minibag to floor: 5 min

Total: 53 min.

## Abbreviations

ACEI: Angiotensin converting enzyme inhibitor
AIDS: Acquired immune deficiency syndrome
APACHE: Acute Physiology and Chronic Health Evaluation
ARB: Angiotensin II receptor blocker
ASA: Acetylsalicylic acid
CNST: Coagulase-negative *Staphylococci*
cSSTI: Complicated skin and soft tissue infection
EPR: Electronic Patient Record
HCTZ: Hydrochlorothiazide
HDR: Health Data Records
HIV: Human immunodeficiency virus
ICU: Intensive Care Unit
MCS: Monte Carlo Simulation
MRSA: Methicillin-resistant *Staphylococcus aureus*
NSAID: Nonsteroidal anti-inflammatory drug
PPI: Proton pump inhibitor
RIFLE: Risk, Injury, Failure, Loss of kidney function, and End-stage kidney disease
SD: Standard deviation
SHSC: Sunnybrook Health Sciences Centre
SPIRIT: Stewardship Program Integrating Resource Information Technology
SSTI: Skin and soft tissue infection
TNF-α: Tumor necrosis factor alpha
UTI: Urinary tract infection
VRE: Vancomycin-resistant *Enterococci*
